# Emotional Control among Nurses against Work Conditions and the Support Received during the SARS-CoV-2 Pandemic

**DOI:** 10.3390/ijerph18179415

**Published:** 2021-09-06

**Authors:** Iwona Malinowska-Lipień, Tadeusz Wadas, Joanna Sułkowska, Magdalena Suder, Teresa Gabryś, Maria Kózka, Agnieszka Gniadek, Tomasz Brzostek

**Affiliations:** 1 Institute of Nursing and Midwifery, Faculty of Health Sciences, Jagiellonian University Medical College, 31-501 Kraków, Poland; magdalena23.suder@student.uj.edu.pl (M.S.); ter.gabrys@uj.edu.pl (T.G.); maria.kozka@uj.edu.pl (M.K.); agnieszka.gniadek@uj.edu.pl (A.G.); tomasz.brzostek@uj.edu.pl (T.B.); 2 Małopolska District Chamber of Nurses and Midwives, 31-153 Kraków, Poland; twadas@moipip.org.pl; 3 Faculty of Medicine, Jagiellonian University Medical College, 31-501 Kraków, Poland; joasia.sulkowska@doctoral.uj.edu.pl

**Keywords:** emotional control, nurses, anger, fear, depression, COVID-19

## Abstract

Introduction. Working in the state of a pandemic is a huge mental load for the medical environment. Aim. Evaluation of emotional control among nurses against work conditions and the support received during the SARS-CoV-2 pandemic. Material and methods. The research was performed among nurses (*n* = 577) working during the pandemic caused by the SARS-CoV-2 virus in infectious (*n* = 201) and non-infectious (*n* = 376) wards in 11 Polish hospitals. To evaluate work conditions, the questionnaire prepared by the authors and the Emotional Control Scale (Courtauld Emotional Control Scale—CECS), which rates the control of anger, depression, and fear were used. Results. In the entire research group, fear had the highest rate of suppression among the negative emotions—18.25 points, 17.91 points in infectious wards and 18.44 points among nurses working in non-infectious wards; *p* > 0.05. The nurses fear was significantly repressed when there was no possibility of the nurses having to perform a COVID-19 test in the workplace; *p* < 0.05. A larger emotional supressed occurred in nurses who simultaneously declared the perception of increased stress level; *p* < 0.05. Conclusions. A high level of emotion suppression, especially regarding fear, combined with higher stress levels, occurring irrespective of the ward, points at the need for mental support for the researched nurses.

## 1. Introduction

The pandemic caused by the SARS-CoV-2 virus impacts both the physical and mental condition of the infected persons themselves as well as those providing care for them [1]. Healthcare employees who have direct contact with infected patients are especially susceptible. Reports from all over the world inform us on the growing exhaustion experienced by medical personnel and physical discomfort related to long working hours in protective overalls and masks as well as fear of infection [2,3]. Among medical care employees, nurses are especially subjected to the above loads [4,5]. The developing COVID-19 pandemic exercises a huge pressure on the work of nurses. A work environment where expectations are high, where there is an extreme physical and mental load, where there is a lack of time and social support may lead to the accumulation of work stress, which, in turn, causes fear, post-traumatic stress disorder, and professional burnout as well as mental and health problems [6,7,8]. In situations where negative emotions are experienced, it is important to evaluate emotional control, which constitutes a subjective conviction of an entity about their ability to control their reactions [9]. Emotional control depends on the ability to make decisions and conviction regarding the validity of the choice. While expressing emotions, it is one’s personal resources that are important, namely a sense of control, self-effectiveness, resourcefulness, optimism, valuation, and coping with stressing events. Excessively held and long-lasting negative emotions may lead to nervous disorders and psychosomatic illnesses. Depending on the situation, negative emotions may motivate actions or contribute to lack of will to take them [10,11]. Suppression, i.e., inhibiting the expression of emotions, leads to their intensification or, in the case of their long-term maintenance, leads to emotional tension. Unexpressed emotions adversely affect health. According to psychologists, they become the basis of numerous neurotic disorders and psychosomatic diseases. Expressing negative emotions is beneficial and recommended in psychotherapy [7,9,10].

Working in the conditions of the pandemic is a huge load for the medical environment [1]. It requires close cooperation, a professional attitude, and solidarity from the nursing personnel. The feeling of responsibility for the health of patients, themselves, and other team members as well as for the people close to them motivates the strict observance of procedures and critical thinking to provide safety for themselves and others [12,13].

So far, no results of studies comparing emotions experienced by nurses working in the infectious and non-infectious ward have been found. However, there are many studies that have shown that nurses working in infectious disease departments during the COVID-19 pandemic experience physical fatigue and mental stress. Additionally, these nurses felt that they had experienced injustice and expressed dissatisfaction with the unequal exposure that they had to the infectious environment compared to medical staff working in other departments. They experienced a range of negative mental and emotional reactions, including stress [14,15,16], anxiety [15], and depression [17].

Our study is one of the first of its kind and concerns the emotions felt by nurses working in hospital wards in Poland during the pandemic. All hospital employees are always threatened by a risk of infection; however, the scope of this risk during the pandemic depends on the type of ward, and this risk is not evenly distributed. The staff working in the emergency, intensive care unit, infectious wards or those areas converted into such places are possibly at a higher risk than other staff. Due to that reason, our research focuses on nurses employed in high-risk wards, namely infectious wards or those converted into infectious wards for patients with COVID-19.

## 2. Materials and Methods

### 2.1. Aim of Research

The aim of the research was to evaluate emotional control among nurses against their work conditions and the support received during the COVID-19 pandemic caused by the SARS-CoV-2 virus.

### 2.2. Design

This was a cross-sectional study of hospital nurses working in the Małopolska region of Poland.

### 2.3. Setting

The participants worked in infectious wards and in non-infectious wards.

### 2.4. Instrument

This research used the diagnostic polling method with a questionnaire designed by the authors for this research and with the Courtauld Emotional Control Scale (CECS), which was developed by M. Watson and S. Greer and adapted by Z. Juczyński [9]. The reliability (Cronbach’s alpha) of the Polish version of the CECS Scale was 0.80 for anger control; for depression control—0.77; fear control—0.78, and for the general coefficient of emotion control (CECS)—0.87. The questions in the author questionnaire concerned socio-demographic and professional data (including gender, age, marital status, education, and work experience). The other questions were related to work conditions, i.e., determining procedures for actions to be taken towards a patient suspected of or who has been diagnosed with COVID-19 infection; training on putting on and taking off protective gear; supplies with personal safety measures; the possibility being tested for COVID-19 in the work place (i.e., nasopharynx swab and marking with the RT-PCR method or antigen test); support from the employer, psychologist, and also co-workers; and work load. The questions also concerned the stress level felt due to the pandemic, the evaluation of nursing staff protection during the pandemic, the necessity to work overtime, and the number of shift hours. The research used the Courtauld Emotional Control Scale (CECS). This tool contains 21 entries that are divided into 3 subscales. Each of them contains seven statements that concern the manner of showing anger, depression, and fear. The scale is designed to poll adults, both healthy ones and patients, and it serves to measure subjective anger, fear, and depression control in difficult life situations. A respondent, while checking the suitable answer, estimates the how frequently they express emotions based on the ways listed in the questionnaire on a 4-grade scale from “hardly ever”—1 point to “almost always”—4 points. For each of the subscales, results are computed separately. The sum of the results in each of the subscales falls within 7–28 points. After summing the results of all three subscales, a general coefficient of emotional control is obtained, which determines the researched person’s conviction about their ability to control their reactions in a situation where they are experiencing negative emotions. The total coefficient fits within the range of 21–84 points. The higher the value of the coefficient, the larger the suppression of negative emotions [9]. The Courtauld Emotional Control Scale (CECS) was used in the version in accordance with the Polish adaptation by Juczyński [9].

### 2.5. Sample, Recruitment, and Data Collection

The size of the trial was calculated using the method of covariance structure modelling [18], and the minimum size of the trial for our study was 377. The minimum size of the group was calculated by assuming the size of the general group—19,716; the estimated fraction size (*p*)—50%; the significance level (α)—5% (0.05); and the permissible error (e)—5%. A total of 706 nurses out of the 19,716 nurses registered in the Malopolska Region Chamber of Nurses and Midwives (Polish: MOIPiP) (data as of 1 August 2021) agreed to voluntarily participate in the study. Of the original 706, 577 of them were qualified to undergo the analysis. The study covered nurses employed in 11 out of 30 public hospitals in this region of Poland. The researched nurses were divided into two groups: those working in infectious wards (*n* = 201) and those working in non-infectious wards (*n* = 376). The group of nurses working in infectious wards also included nurses employed in wards converted into infectious ones, emergency departments, and intensive care units that have contact with COVID-19 patients. The group of nurses working in non-infectious wards consisted of nurses working in hospital wards where they did not provide care for patients with COVID-19. The research was performed using an online questionnaire, which was only available to the nurses. It was anonymous and voluntary. The nursing staff was recruited through the Malopolska Region Chamber of Nurses and Midwives website (MOIPiP), and the link to the questionnaire along with the cover letter was sent to the representatives of the Chamber working in healthcare facilities. The inclusion criteria for this study were as follows: 1/consent to participate in the research; 2/being a nurse working in the hospital during the pandemic, i.e., from March 2020. The exclusion criteria were: 1/a lack of consent for the research; 2/being a nurse working in healthcare facilities other than a hospital; 3/having no active job as a nurse (i.e., retired/disabled/inability to perform the job/suspended right to perform the job/maternity/childcare leave); 4/no job activity after March 2020, i.e., from the moment of the pandemic announcement in Poland. Complete responses were provided by 706 nurses. A total of 129 forms were excluded from the analysis, 125 of which were excluded due to the nurses working in facilities other than a hospital as well as 4 forms from nurses who completed the questionnaire but did not check the option to consent to the participation in the research. Finally, 577 forms were subjected to the analysis.

The research was approved by the Bioethical Commission of Jagiellonian University (approval KBEUJ) No. 1072.6120.346.2020. The study was performed from 20 December 2020 to 28 February 2021.

### 2.6. Statistical Analysis

The analysis was performed using the TIBCO STATISTICA 13.3 software package (StatSoft, Inc., Tulsa, OK, USA). The results of the study concerning work conditions and support were presented in the form of frequency and percentage values, while the results of the Emotion Control Scale (CECS) and each subscale of the CECS were demonstrated as mean values (x) and standard deviation (SD). For each respondent, mean results within each CECS subscale were computed separately. The analysis of significance of the differences between the mean values in the compared groups was performed in observance wit the rules of the chosen test. The spread of the researched quantitative variables was checked using Kołmogorow–Smirnow tests, the group variance equalities were evaluated using Levene’ or F Fisher–Snedeckor tests. To perform the analysis, one-way multi-dimensional analysis of variances was used; if the result of the one-way MANOVA was statistically significant, the one-way ANOVA analysis was performed with Tukey post hoc tests. In all of the analyses, the results were accepted as significant in cases when the probability value *p* was smaller than the accepted significance level 0.05 (*p* < 0.05).

## 3. Results

Most of the study participants were women (*n* = 560; 97.05%). A total of 201 of the nurses were employed in infectious wards, and the remaining nurses worked in non-infectious wards (*n* = 376). The ages of the researched nurses ranged from 22 to 66 years of age, and the average age was 41 years (SD = 11.84). The highest percentage of the research group were nurses between 41 and 50 years of age (31.89%). The research group largely consisted of married nurses (*n* = 391; 67.76%). Most of the group held a university degree: Master of Science in Nursing—46.62% (*n* = 269), Bachelor of Science in Nursing—31.54% (*n* = 182). Of the group, 47.04% (*n* = 273) declared over 20 years of working experience; Table 1.

The overall result of the Courtauld Emotional Control Scale (CECS) in the entire group of evaluated nurses was 54.28 pts (SD = 11.78). The level of emotion suppression among nurses working in infectious wards equalled 54.11 pts (SD = 12.23), which is similar to the results obtained for the nurses from non-infectious wards—54.37 pts (SD = 11.35); *p* = 0.865. The largest suppression of negative emotions concerned fear—the research group obtained a mean result at the level of 18.25 pts (SD = 4.45), in which nurses from infectious wards score17.91 pts (SD = 4.75), while in non-infectious wards score18.44 pts (SD = 4.28); *p* = 0.299. Regarding anger suppression, the research group obtained a mean result of 18.09 pts (SD = 4.89), where those working in infectious wards scored 18.21 pts (SD = 4.96) and those working in non-infectious wards score18.03 pts (SD = 4.86); *p* = 0.728. As for depression suppression, the research group scored 17.93 pts (SD = 4.58), where those working in infectious wards obtained 17.98 pts (SD = 4.66), and those working in in non-infectious wards score 17.90 pts (SD = 4.54); *p* = 0.773.

A significant dependency within emotional control in reference to gender was determined in the entire research group—women much more frequently suppressed negative emotions on the anger scale compared to men (*p* = 0.026). In a similar way, women working in non-infectious wards suppressed their anger significantly more than men working in the same type of wards (*p* = 0.046). The study demonstrated a correlation between the age of the studied nurses and the level of depression suppression (*p* = 0.025); among the participants, the youngest and oldest nurses suppressed emotions significantly more often, i.e., nurses aged 22–30 and 51–66 years of age, respectively, Table 2. Post hoc tests did not determine significant correlation between other socio-demographic data, type of ward, and the emotional control coefficient (CECS); *p* > 0.05.

The analysis of work the conditions of the hospital staff working during the pandemic showed that in the majority of cases, the wards determined procedures for how to deal with a COVID-19-infected patient (*n* = 545; 94.45%): 94.02% in infectious wards (*n* = 189) and 94.42% in non-infectious wards (*n* = 356). The staff also held the opinion (*n* = 354; 61.35%) that they had received training on how to deal with a COVID-19-infected patient or one who suspected of the infection as well as training concerning putting on/taking off protective clothing; affirmative answers regarding training were71.64% in infectious wards (*n* = 144) and 55.74% in non-infectious ones (*n* = 210). Most of the research group indicated (*n* = 401; 69.50%) that the hospitals provided them with personal safety measures, 80.10% (*n* = 161) and 63.82% in infectious and non-infectious wards, respectively (*n* = 240). The possibility to freely use personal safety measures was indicated by 67.07% (*n* = 387) nurses, with 80.10% of the nurses in infectious wards attesting to this fact (*n* = 158) and 60.90% in non-infectious ones (*n* = 229). Of the nurses, 53.37% (*n* = 308) had the possibility to freely perform a COVID-19 test on themselves in the workplace, which was indicated 59.20% of the nurses in infectious wards (*n* = 119) and 50.26% of nurses in non-infectious wards (*n* = 189). A higher workload during the pandemic was demonstrated by 85.44% (*n* = 493) of nurses, with 91.54% of nurses in infectious wards (*n* = 184) and 82.18% of nurses in non-infectious wards (*n* = 309) attesting to this. The perception of higher stress intensity in relation to the pandemic was indicated by 97.22% (*n* = 561) of the nurses from the research group, with 97.51% of the nurses in infectious wards (*n* = 196) and 97.07% of nurses in non-infectious wards (*n* = 365) attesting to this. A total of 85.09% (*n* = 491) of the nurses indicated a staff deficiency in hospital wards, which was more specifically indicated by 86.06% of the nurses in infectious wards (*n* = 173) and 84.57% of the nurses in non-infectious wards (*n* = 318). With the reference to the pandemic, over a half of the research group (*n* = 340; 58.92%) worked overtime, respectively 72.63% o (*n* = 146) and 51.59% of the nurses working in infectious and non-infectious wards (*n* = 194), respectively. During the pandemic nurses mostly served 12-h shifts (*n* = 371; 64.30%), with 52.23% of the nurses working in infectious wards (*n* = 105) and 70.74% of the nurses in non-infectious wards (*n* = 266) attesting to this fact. In the whole research group, a significant correlation was found between emotional suppression and the possibility of undergoing a swab test in the workplace. Nurses much more frequently suppressed negative emotions on the fear subscale when they had no possibility of performing a swab test (*p* = 0.035); nurses working in non-infectious wards also showed this dependency (*p* = 0.020). The analysis of the research indicated that higher emotional suppression occurred in nurses who declared a higher stress perception; *p* = 0.041. Post hoc tests showed that no relationship between work conditions and the type of ward with anger/depression/fear suppression and the general emotional control coefficient (CECS) was demonstrated; *p* > 0.05. The studied group of nurses could mainly count on the support of co-workers (*n* = 522; 90.46%), with 94.02% of the staff working in infectious wards (*n* = 189) and 88.56% of the staff working in non-infectious ones (*n* = 333) attesting to this. A total of 55.11% (*n* = 318) could rely on their employer’s support, with 60.20% (*n* = 121) and 52.39% of nurses from infection and non-infectious wards (*n* = 197) reporting this. In turn, psychological counselling was available to 27.20% (*n* = 157) of nurses, with 29.85% of the staff working in infectious wards (*n* = 60) and 25.79% of the staff working in non-infectious wards (*n* = 97) indicating this, Table 3. Post hoc tests showed that there was no relationship between the type of support obtained or type of ward with anger/depression/fear suppression and the general emotional control coefficient (CECS) was indicated; *p* > 0.05.

## 4. Discussion

Our research using the Courtauld Emotional Control Scale (CECS) demonstrates the inclination of the research group of nurses to suppress negative emotions. The nurses obtained average scores within the emotional control level (CECS), which is understood as a subjective conviction of an entity about their ability to control their emotional reactions through the suppression or external expression of responses to difficult situations [9]. The mean value of the overall coefficient of emotional control intensity (CECS) was 54.28, and mean values within the range of the anger/depression/fear (18.09/17.93/18.25) suppression subscales were higher than the norms obtained by Juczyński for the Polish population in the period of time before the pandemic (CECS-49.97; anger-16.01; depression-16.88; fear—17.08) [9]. They were, however, in alignment with the research by Bidzan et al., which was performed in Poland in March 2020, right after the announcement of the pandemic [19]. The research by Bidzan et al. was conducted on a group of healthcare employees (including nurses), and it aimed to determine their emotional control level (CECS) related to the COVID-19 pandemic. Both the current research and the research by Bidzan et al. indicate that nurses adopt suppression mechanisms as a way to cope with negative emotions caused by work in the conditions of the pandemic [19]. These results are alarming because the suppression of emotional expression, especially in the state of the widespread threat of a pandemic, leads to their intensification or triggers long-term effects in the form of emotional tension, consequently posing a health threat [20]. Unexpressed emotions may contribute to neurotic disorders and psychosomatic diseases. The results of our research demonstrated that nurses who worked in the hospital, both in infectious and non-infectious wards, felt strong emotional reactions, suppressed their emotions, and focussed on task completion. In their research, Aliakbari et al. [21] proved that during unexpected cataclysms or pandemics, nurses often disregarded their own health and safety and that they worked selflessly due to their strong ethics and professionalism so as to cope with the situation. Although our research research did not demonstrate any influence of the support received by the nurses on their emotional control, it is especially crucial to pay attention to the insufficient support from psychologists—only 27.20% of the research group could count on such support. A total of 90% of the researched nurses could, however, rely on support from their co-workers, and a half could rely on support from their employers of supervisors. Meanwhile, it seems obvious that nurses working under stress resulting from the pandemic threat should be granted unlimited access to psychological counselling. Studies by other authors prove that the intensification of mental disorders was larger among staff with more limited access to education and psychological support. Properly organised training sessions and activities in the hospital helped reduce stress, while the psychological counsellors present in hospitals provided due support [12,22,23].

Emotional control and management play a decisive role in healthcare and the occurrence of psychological disorders [24]. In the state of the pandemic, emotional control is accompanied by the increase of fear and anger levels [20]. In the current study, the highest suppression of negative emotions concerned fear, and in nurses working in infectious wards—anger and depression. Our research demonstrated that performing a COVID-19 test in the workplace was a factor resulted in an increase in fear expression. This dependency was demonstrated for the overall research group, and it was also demonstrated separately in the group of nurses working in non-infectious wards. Moreover, the current research observed that people who perceived a higher intensity of stress due to the pandemic indicated a higher level of emotion suppression. In the research performed in China by Lai J Ma S et al. on 764 nurses and 493 physicians, as many as 50.4% of the participants reported symptoms of depression, 44.6% reported symptoms of fear, and 34.0% reported symptoms of insomnia. The levels of these symptoms were higher in people working with COVID-19-diagnosed patients and in women among nurses [12]. Huang et al. recorded that 23.04% out of 230 employees of an infectious hospital pointed out the occurrence of fear [25]. In the research by Chew et al., which was performed on 180 employees working with COVID-19 patients, the level of social support was substantially related to the feeling of one’s own effectiveness and the quality of sleep, while it was negatively correlated with the levels of fear and stress. The level of fear was significantly related to the stress level, which, in turn, negatively impacted the feelings of one’s own effectiveness and sleep quality [20].

This study demonstrated that women anger suppression strategies more often than men. The result is similar to the one obtained by Ham and You [26]. All over the world, the nursing profession is mostly performed by women, who are expected to stay self-controlled and maintain harmonious relationships. Due to the specifics of the job, nurses often suppress anger, not demonstrating negative emotions while working with a patient. Such situations make nurses unable to manage their demonstrations of anger in a constructive manner, which finally, combined with the lack of ability to cope with difficult situations, leads to stress, exhaustion, and professional burnout [26]. Studies by other authors prove that people who are often subjected to fear as a condition are at risk for it becoming a feature and will contribute to occurrence of anxiety situations (social, somatic, etc.) [27,28]. The COVID-19-related pandemic caused fear and anxiety among people around the world [3,10,13,23].

An important factor determining perceived emotions and the ability to control them is the workplace and workplace conditions. The present research did not demonstrate any direct influence of the workplace on perceived emotions and the ability to control them. Up to date research shows that nurses working in infectious wards managed their care of COVID-19 patients slightly better because due to the fact that they were, working with potentially infected patients on a daily basis, they were, in a way, used to the specificity of this job. The necessity for the staff of other-turned-infectious wards to quickly adjust to the changed procedures required mobilisation, the extension of knowledge and skills, and was related to the exposure of higher stress level [29,30]. In the present research, it was this group who presented a lower fear intensity, though the level of anger suppression was the highest in this group of nurses.

Most of the nurses participating in our research felt that there was a staff deficiency in their workplaces during the COVID-19 pandemic and that they worked beyond their scheduled hours, and one fifth worked in the 24 h-shift system. A growing number of infections among the staff and self-isolation due to the contact with infected people caused problems with manning nursing shifts, and the remaining nursing staff, serving overtime shifts, reported a higher workload. Xiao H and Zhang Y et al. demonstrated that in the state of pandemic, a change to the working hours and frequent overtime result in higher fear and depression levels among medical personnel, which impacts their daily lives as well as their ability to sleep and their possibility to receive rest [31]. The study by Chew et al. indicated the increase in the stress/fear/depression levels and negative physical symptoms in healthcare workers during the pandemic [20].

The problem of Polish hospitals being quickly filled up with patients, which was visible during the second wave of the pandemic, was that the deficiency of the staff led to the occurrence of negative emotions in the group of medical personnel. The appearance of difficulties in the workplace may result in the increase of tension and anxiety among the employees in Poland. The more a certain person perceives a given event as a threat, the more intense the anxiety is. That is why the answer to the question of how to reduce the anxiety level of healthcare personnel at work in an atypical environment is becoming even more important, as is the question of how to influence work in such conditions. The global course of events related to the COVID-19 pandemic and the safety of the environment where healthcare employees work has become a crucial issue, both physical and emotional. Our study suggests that the provision of psychological counselling and the possibility to perform COVID-19-aimed tests may contribute to the decrease of adverse emotion suppression.

### Study Limitations

The presented studies are not free of limitations due to the fact that they were performed during another wave (the third one) of the pandemic in Poland; thus, it may be assumed that the nursing personnel had managed to elaborate certain work methods to deal with COVID-19-infected or patients suspected of infection. That is why in most cases, emotional control was unaffected by work conditions or received support, but why it could be influenced by the daily routine. No correlation between difficult working conditions during the pandemic and emotional control can be understood as evidence for the nursing staff’s mobilisation and adjustments to the current situation. It is probable that such a study performed at the beginning of the pandemic or outside of the current wave of infections would produce different results. Any planned future research should be more complex and of longitudinal manner. Such research should incorporate a few time points, which might help study the connection between the incentive that nurses have to suppress their emotions and working conditions and support. The small percentage of men in the research group requires an analysis in a study with more male participants. In addition, subsequent studies deem it interesting to investigate how the variables regarding the work environment during the COVID-19 pandemic and the hospital environment are related and which factors are important in suppressing emotions in Polish nurses.

## 5. Conclusions

A high level of emotional suppression e, especially regarding the feeling of fear, combined with higher stress level, occurring irrespective of the ward, points to the need of mental support for the researched nurses. The ability to perform a COVID-19 test to verify SARS-CoV-2 infection in the workplace can help to reduce anxiety suppression.

## Figures and Tables

**Table 1:** Research group profile.

		Nurses Working in the Infectious Diseases Ward (*n* = 201)		Nurses Working in the Non-Infectious Ward (*n* = 376)		All Study Nurses (*n* = 577)	
		*n*	%	*n*	%	*n*	%
Gender, *n* (%)							
	Women	196	97.51	364	96.80	560	97.05
	Men	5	2.49	12	3.20	17	2.95
Age, *n* (%)							
	22–30 years	60	29.85	107	28.46	167	28.94
	31–40 years	28	13.93	57	15.16	85	14.73
	41–50 years	72	35.82	112	29.79	184	31.89
	51–66 years	41	20.40	100	26.60	141	24.44
Marital status, *n* (%)							
	Married	136	67.66	255	67.82	391	67.76
	Single	32	15.92	71	18.88	103	17.85
	Informal relationship	24	11.94	31	8.24	55	9.53
	Divorced/Separated	8	3.98	9	2.39	17	2.95
	Widowed	1	0.50	10	2.66	11	1.91
Education, *n* (%)							
	Medical secondary education	27	13.43	55	14.63	82	14.21
	Bachelor degree in nursing	63	31.34	119	31.65	182	31.54
	Master degree in nursing	99	49.25	170	45.21	269	46.62
	Higher education, degree obtained in a faculty other than nursing	111	5.48	31	8.24	42	7.28
	Nurse with Ph.D. degree	1	0.50	1	0.27	2	0.35
Seniority, *n* (%)							
	<year	6	2.99	10	2.66	16	2.77
	from 1–5 years	53	26.37	94	25.00	147	25.48
	from 6–10 years	19	9.45	35	9.31	54	9.36
	from 11–20 years	28	13.93	59	15.69	87	15.08
	from 21–30 years	61	30.35	95	25.27	156	27.04
	over 30 years	34	16.92	83	22.07	117	20.28

Note: *n*—number.

**Table 2:** Controlling emotions of the surveyed nurses depending on sociodemographic data.

	Anger Suppression			Depression Suppression			Fear Suppression			General Coefficient of Emotional Control (CECS)		
	Infectious Diseases Ward	Non-Infectious Ward	*p*-Value	Infectious Diseases Ward	Non-Infectious Ward	*p*-Value	Infectious Diseases Ward	Non-Infectious Ward	*p*-Value	Infectious Diseases Ward	Non-Infectious Ward	*p*-Value
	Mean (SD)			Mean (SD)			Mean (SD)			Mean (SD)		
Gender												
women (*n* = 560)	18.28 (4.92)	18.11 (4.83)	**0.026**	18.09 (4.58)	17.90 (4.54)	0.343	17.90 (4.71)	18.41 (4.31)	0.786	54.28 (12.07)	54.43 (11.59)	0.293
men (*n* = 17)	15.60 (6.27)	15.58 (5.19)		13.60 (4.83)	17.75 (4.53)		18.20 (6.61)	19.25 (3.04)		47.40 (17.81)	52.58 (10.50)	
*p*-value	0.310	**0.046**		0.121	0.900		0.916	0.706		0.503	0.426	
Age												
22–30 years (*n* = 167)	18.76 (5.01)	18.60 (5.21)	0.254	18.10 (4.59)	18.41 (4.43)	**0.025**	18.26 (4.68)	18.90 (4.59)	0.338	55.13 (11.59)	55.92 (11.78)	0.071
31–40 years (*n* = 85)	17.42 (5.45)	18.11 (4.35)		16.75 (4.65)	16.72 (4.35)		16.10 (4.18)	18.13 (4.28)		50.28 (11.18)	52.98 (10.51)	
41–50 years(*n* = 184)	18.15 (4.95)	17.27 (4.59)		17.88 (4.37)	17.72 (4.86)		18.13 (4.47)	18.29 (3.68)		54.18 (11.82)	53.28 (11.18)	
51–66 years (*n* = 141)	18.07 (4.63)	18.21 (4.99)		18.82 (5.22)	18.24 (4.31)		18.21 (5.50)	18.28 (4.57)		55.12 (13.85)	54.75 (12.23)	
*p*-value	0.697	0.205		0.269	0.092		0.114	0.743		0.308	0.158	
Marital status												
Married (*n* = 391)	18.23 (4.91)	18.02 (4.69)	0.476	17.84 (4.68)	17.90 (4.46)	0.219	17.61 (4.73)	18.50 (3.97)	0.312	53.68 (12.43)	54.44 (10.98)	0.273
Single (*n* = 103)	19.21 (4.87)	18.31 (5.37)		18.87 (4.74)	18.09 (4.32)		17.90 (4.85)	18.46 (4.92)		56.00 (12.65)	54.87 (12.48)	
Informal relationship (*n* = 55)	17.37 (5.67)	18.41 (5.06)		18.12 (4.63)	18.03 (5.41)		19.58 (4.97)	18.64 (4.69)		55.08 (11.66)	55.09 (13.10)	
Divorced/separated (*n* = 17)	16.37 (3.73)	15.12 (4.64)		16.37 (4.72)	14.25 (5.14)		17.87 (3.75)	15.37 (5.83)		50.62 (9.84)	44.75 (14.09)	
Widowed (*n* = 11)	20.00 (3.01)	17.33 (5.00)		18.00	19.55 (4.00)		19.00	19.11 (3.98)		57.00	56.00 (9.68)	
*p*-value	*p* = 1.000	*p* = 0.599		*p* = 1.000	0.296		1.000	0.170		1.000	0.306	
Education *n* (%)												
Medical secondary education (*n* = 82)	18.03 (4.85)	18.07 (4.94)	0.843	19.37 (4.28)	17.16 (5.25)	0.929	19.29 (4.65)	18.18 (5.13)	0.374	56.70 (11.65)	53.43 (13.89)	0.738
Bachelor degree in nursing (*n* = 182)	18.47 (4.95)	17.72 (4.27)		17.85 (4.72)	17.97 (3.90)		18.17 (4.53)	17.89 (3.88)		54.50 (12.37)	53.59 (9.83)	
Master degree in nursing (*n* = 269)	18.43 (4.87)	18.13 (5.17)		18.04 (4.37)	17.95 (4.74)		17.61 (4.68)	18.86 (4.24)		54.09 (11.39)	54.94 (11.84)	
Higher education, degree obtained in a faculty other than nursing (*n* = 42)	16.40 (5.87)	18.25 (5.90)		15.60 (6.53)	18.70 (4.68)		16.90 (6.03)	17.00 (0.3)		48.90 (17.40)	56.29 (12.430)	
Nurse with Ph.D. degree (*n* = 2)	12.00	17.00		18.00	19.00		13.00	18.00		43.00	54.00	
*p*-value	0.333	0.926		0.634	0.898		0.168	0.051		0.236	0.822	
Seniority												
<year (*n* = 16)	17.50 (6.02)	19.80 (6.05)	0.176	17.00 (5.73)	19.20 (3.39)	0.566	18.66 (5.32)	19.90 (4.28)	0.860	53.16 (14.83)	58.90 (11.92)	0.232
1–5 years (*n* = 147)	19.07 (4.88)	18.78 (4.86)		17.98 (4.64)	18.31 (4.28)		17.62 (4.72)	18.89 (4.32)		54.67 (11.30)	56.00 (10.60)	
6–10 years (*n* = 54)	18.00 (5.99)	16.97 (5.45)		18.16 (4.93)	16.57 (5.28)		18.05 (5.71)	17.88 (5.14)		54.21 (14.41)	51.42 (13.87)	
11–20 years (*n* = 87)	16.64 (4.82)	17.71 (4.32)		16.61 (4.45)	17.40 (4.50)		16.75 (4.06)	18.50 (4.03)		50.00 (11.47)	53.61 (10.44)	
21–30 years (*n* = 156)	18.36 (4.99)	17.57 (4.61)		17.86 (4.65)	17.81 (4.83)		18.29 (4.46)	17.99 (3.68)		54.52 (12.33)	53.36 (11.44)	
>30 years (*n* = 117)	18.17 (4.32)	18.14 (5.04)		19.41 (4.49)	18.30 (4.26)		18.41 (5.26)	18.46 (4.63)		56.00 (12.45)	54.90 (12.21)	
*p*-value	0.424	0.293		0.685	0.161		0.650	0.812		0.484	0.208	

Note: SD—standard deviation; *p*-values < 0.05; Statistically significant differences have been marked in bold.

**Table 3:** Emotional control of the surveyed nurses depending on working conditions and depending on the support received during the COVID-19 pandemic.

	Suppress Anger			Suppress Depression			Suppression of Anxiety			General Emotional Control Index (CECS)		
	Infectious Diseases Ward	Non-Infectious Ward	*p*-Value	Infectious Diseases Ward	Non-Infectious Ward	*p*-Value	Infectious Diseases Ward	Non-Infectious Ward	*p*-Value	Infectious Diseases Ward	Non-Infectious Ward	*p*-Value
	Mean (SD)			Mean (SD)			Mean (SD)			Mean (SD)		
Working conditions during a pandemic												
COVID-19 procedures developed in the ward												
Yes (*n* = 545)	18.41 (4.89)	17.97 (4.86)	0.584	18.08 (4.63)	17.88 (4.54)	0.681	17.91 (4.71)	18.40 (4.21)	0.867	54.41 (12.18)	54.26 (11.43)	0.852
No (*n* = 15)	15.11 (4.07)	19.33 (6.15)		16.44 (4.97)	18.00 (5.29)		17.00 (5.24)	20.66 (5.78)		48.55 (11.70)	58.00 (16.91)	
I don’t know (*n* = 17)	15.00 (8.66)	18.92 (4.29)		16.00 (6.00)	18.38 (4.44)		20.66 (6.50)	18.53 (5.28)		51.66 (17.03)	55.84 (12.71)	
*p*-value	0.087	0.678		0.410	0.994		0.590	0.704		0.456	0.833	
Organised training in treating a COVID-19 patient, as well as dressing and undressing of protective clothing												
Yes (*n* = 354)	18.02 (4.91)	18.11 (4.78)	0.872	17.86 (4.63)	17.81 (4.29)	0.437	17.95 (4.45)	18.41 (3.92)	0.065	53.85 (11.68)	54.35 (11.09)	*p* = 0.377
I don’t know (*n* = 23)	16.00 (7.58)	18.05 (4.76)		16.20 (6.05)	17.21 (4.73)		14.20 (5.67)	16.57 (3.82)		46.40 (18.28)	51.84 (10.87)	
No, I had to learn everything myself (*n* = 199)	18.96 (4.81)	17.91 (5.01)		18.48 (4.64)	18.14 (4.86)		18.13 (5.36)	18.76 (4.74)		55.57 (13.02)	54.82 (12.27)	
*p*-value	0.356	0.904		0.536	0.681		0.322	0.155		0.345	0.729	
Sufficient provision of personal safety means in the ward												
Yes (*n* = 401)	18.15 (5.08)	18.17 (4.72)	0.542	17.76 (4.70)	17.66 (4.34)	0.198	17.89 (4.71)	18.34 (4.12)	0.531	53.81 (12.42)	54.18 (10.99)	*p* = 0.838
No (*n* = 176)	18.47 (4.47)	17.78 (5.10)		18.87 (4.46)	18.31 (4.85)		17.97 (4.91)	18.62 (4.55)		55.32 (11.48)	54.71 (12.49)	
*p*-value	0.631	0.366		0.203	0.402		0.751	0.738		0.481	0.814	
Possibility to use personal safety means freely, without limitations												
Yes (*n* = 387)	18.00 (5.10)	18.22 (4.77)	0.762	17.96 (4.73)	17.71 (4.39)	0.669	17.91 (4.82)	18.45 (4.19)	0.750	53.88 (12.42)	54.39 (11.22)	*p* = 0.888
No (*n* = 190)	19.00 (4.33)	17.72 (4.99)		18.04 (4.44)	18.18 (4.76)		17.90 (4.49)	18.43 (4.42)		54.95 (11.60)	54.35 (12.08)	
*p*-value	0.257	0.335		0.909	0.533		0.829	0.990		0.481	0.814	
Possibility to perform a COVID-19 test at the workplace												
Yes, without problems (*n* = 308)	18.00 (4.75)	17.79 (4.91)	0.236	17.71 (4.84)	17.42 (4.34)	0.119	17.67 (4.79)	18.02 (4.01)	**0.035**	53.39 (12.24)	53.24 (11.26)	0.107
Yes, but I had to struggle for this (*n* = 114)	18.39 (5.51)	17.43 (4.36)		18.62 (4.43)	17.95 (4.85)		17.86 (5.05)	18.85 (4.42)		54.88 (12.60)	54.25 (11.50)	
No, because there was no permission from the employer (*n* = 142)	18.87 (5.19)	18.73 (5.02)		18.25 (4.25)	18.70 (4.60)		18.80 (4.11)	18.81 (4.60)		55.93 (11.65)	56.25 (11.98)	
No, because there is no swab point (*n* = 13)	17.87 (4.64)	19.50 (4.96)		17.50 (5.15)	17.33 (4.80)		18.25 (5.11)	19.83 (4.02)		53.62 (13.59)	56.66 (10.81)	
*p*-value	0.887	0.184		0.716	0.119		0.418	**0.020**		0.760	0.165	
Work load												
Yes (*n* = 493)	18.16 (5.00)	18.02 (4.92)	0.447	17.96 (4.67)	18.05 (4.58)	0.444	17.84 (4.76)	18.48 (4.35)	0.973	53.98 (12.21)	54.56 (11.99)	0.490
Hard to evaluate (*n* = 63)	19.63 (3.64)	18.41 (4.85)		18.27 (4.54)	17.56 (4.00)		19.63 (3.85)	18.37 (3.53)		57.54 (10.48)	54.35 (9.48)	
No (*n* = 21)	17.16 (6.01)	16.81 (3.54)		18.00 (5.40)	16.12 (5.22)		16.66 (5.68)	17.87 (5.16)		51.83 (16.30)	50.81 (8.59)	
*p*-value	0.117	0.477		0.999	0.314		0.539	0.816		0.674	0.499	
Feeling higher intensity of stress in relation to the pandemic												
Yes (*n* = 561)	18.25 (4.94)	18.12 (4.88)	0.110	18.04 (4.62)	17.98 (4.56)	0.062	17.90 (4.79)	18.48 (4.28)	0.290	54.19 (12.19)	54.59 (11.60)	**0.041**
Hard to evaluate (*n* = 6)	17.50 (2.12)	14.75 (1.25)		16.00 (1.41)	14.75 (1.89)		17.50 (0.70)	20.25 (2.62)		51.00 (2.82)	49.75 (1.70)	
No (*n* = 10)	16.33 (8.08)	15.77 (3.96)		15.66 (9.01)	15.88 (3.68)		18.66 (3.78)	15.88 (4.19)		50.66 (10.40)	47.55 (9.85)	
*p*-value	0.783	0.099		0.559	0.103		0.980	0.125		0.744	0.133	
Nursing staff insufficiency												
Yes (*n* = 491)	18.27 (5.02)	18.05 (4.91)	0.771	17.97 (4.62)	17.95 (4.63)	0.946	17.97 (4.73)	18.47 (4.34)	0.955	54.22 (12.33)	54.48 (11.84)	0.966
Hard to evaluate (*n* = 22)	17.60 (2.40)	17.27 (4.93)		18.60 (4.61)	17.27 (3.84)		18.20 (2.77)	18.16 (3.65)		54.40 (7.63)	52.72 (10.02)	
No (*n* = 64)	17.95 (4.97)	18.14 (4.44)		17.91 (5.15)	17.75 (4.16)		17.34 (5.25)	18.36 (4.06)		53.21 (12.59)	54.26 (9.95)	
*p*-value	0.964	0.805		0.962	0.876		0.977	0.908		0.991	0.955	
Working overtime in relation to the pandemic												
Yes (*n* = 340)	18.28 (4.93)	17.77 (4.96)	0.540	18.26 (4.46)	18.02 (4.65)	0.259	18.19 (4.76)	18.47 (4.09)	0.672	54.73 (11.83)	54.27 (11.69)	0.842
No (*n* = 237)	18.03 (5.06)	18.30 (4.73)		17.25 (5.12)	17.76 (4.43)		17.16 (4.64)	18.40 (4.48)		52.45 (13.20)	54.48 (11.42)	
*p*-value	0.759	0.281		0.209	0.621		0.289	0.916		0.376	0.738	
Number of work hours during the pandemic												
7.35-h shifts (*n* = 48)	18.66 (5.70)	19.15 (4.78)	0.347	17.00 (5.45)	18.15 (4.79)	0.949	15.88 (6.29)	19.41 (3.85)	0.827	51.55 (16.03)	56.71 (11.25)	0.792
12-h shifts (*n* = 371)	18.17 (5.09)	18.21 (4.74)		18.20 (4.69)	17.88 (4.49)		18.05 (4.53)	18.28 (4.36)		54.43 (12.28)	54.39 (11.43)	
12- and 24-h shifts (*n* = 85)	17.36 (4.95)	17.22 (4.89)		17.97 (4.15)	17.75 (4.77)		18.07 (4.59	18.63 (4.48)		53.41 (10.51)	53.61 (12.27)	
24-h shifts (*n* = 71)	19.04 (4.54)	15.92 (5.55)		17.62 (5.01)	17.88 (4.59)		17.84 (5.14)	18.29 (3.75)		54.51 (13.17)	52.11 (12.17)	
>24-h shifts (*n* = 2)	17.00	17.00		20.00	19.00		17.00	18.00		54.00	54.00	
*p*-value	0.454	*p* = 0.101		0.875	0.996		0.757	0.370		0.944	0.459	
Support received during a pandemic												
Support from the employer												
Yes (*n* = 318)	17.69 (4.77)	17.91 (4.71)	0.145	17.76 (4.64)	17.77 (4.31)	0.338	17.62 (4.63)	18.30 (4.11)	0.696	53.08 (11.71)	53.99 (10.87)	0.158
No (*n* = 259)	19.01 (5.15)	18.16 (5.02)		18.32 (4.69)	18.04 (4.79)		18.33 (4.90)	18.59 (4.46)		55.67 (12.89)	54.80 (12.28)	
*p*-value	0.059	0.631		0.281	0.664		0.237	0.985		0.118	0.579	
Counselling of a psychologist												
Yes (*n* = 157)	18.05 (4.72)	18.50 (4.94)	0.432	17.98 (4.33)	17.81 (4.66)	0.800	18.18 (4.79)	18.51 (4.72)	0.433	54.21 (11.80)	54.83 (12.07)	0.736
No (*n* = 420)	18.29 (5.07)	17.86 (4.82)		17.98 (4.81)	17.93 (450)		17.79 (4.73)	18.41 (4.12)		54.07 (12.44)	54.21 (11.38)	
*p*-value	0.895	0.284		0.900	0.851		0.525	0.758		0.920	0.729	
Support from co-workers												
Yes (*n* = 522)	18.19 (4.95)	18.06 (4.83)	0.677	17.96 (4.70)	17.84 (4.54)	0.565	17.90 (4.76)	18.36 (4.28)	0.363	54.05 (12.27)	54.27 (11.55)	0.926
No(*n* = 55)	18.66 (5.31)	17.79 (5.12)		18.33 (4.09)	18.37 (4.57)		18.00 (4.70)	19.02 (4.24)		55.00 (11.98)	55.18 (11.58)	
*p*-value	0.811	0.972		0.687	0.609		0.819	0.419		0.951	0.938	

Note: SD—standard deviation; stat—statistics; *p* -values < 0.05; Statistically significant differences have been marked in bold.

## Data Availability

Not applicable.
